# DrPitA-mediated enrichment of intracellular manganese and phosphate contributes to oxidative stress resistance of *Deinococcus radiodurans*

**DOI:** 10.1128/aem.02107-25

**Published:** 2026-04-21

**Authors:** Zhenming Xie, Shang Dai, Binqiang Wang, Ning Yu, Cheng Huang, Jie Zhao, Chunhui Cai, Furong Zhang, Zichun Tan, Yiting Wang, Yuejin Hua, Bing Tian

**Affiliations:** 1 Institute of Biophysics, College of Life Sciences, Zhejiang University 12377, Hangzhou, China; 2 Hangzhou Institute of Medicine (HIM), Chinese Academy of Sciences 631027, Hangzhou, China; 3 Department of Microbiology, College of Life Sciences, Nanjing Agricultural University 98430, Nanjing, China; 4 State Key Laboratory of Clean Energy Utilization Zhejiang University 441932 , Hangzhou, China; 5 Zhejiang Baima Lake Laboratory Co., Ltd, Hangzhou, China; 6 Cancer Center, Zhejiang University 12377, Hangzhou, China; Washington University in St Louis, St. Louis, Missouri, USA

**Keywords:** *Deinococcus radiodurans*, non-enzymatic antioxidants, oxidative stress, DrPitA, Mn-Pi enrichment

## Abstract

*Deinococcus radiodurans* is an extreme bacterium capable of withstanding ionizing radiation-induced oxidative stress. Non-enzymatic manganese antioxidant complexes play an important role in its resistance to oxidative stress. Here, we demonstrate that the PitA homolog (DrPitA) encoded by *dr0925*, a low-affinity metal-phosphate transporter homolog, mediates enrichment of intracellular manganese-phosphate (Mn-Pi) and participates in the oxidative resistance of *D. radiodurans*. The mutation of *drpitA* significantly reduced intracellular Mn ion and phosphate levels, concomitant with the compromised cell survival under oxidative stress. Under H_2_O_2_ stress, the *drpitA* mutant accumulated excessive reactive oxygen species, resulting in severe protein carbonylation damage compared with the wild type strain. Moreover, the *drpitA* mutant cells exhibit decreased intracellular polyphosphate (PolyP) under H_2_O_2_ stress, indicating that the DrPitA-mediated Mn-Pi enrichment may be related to PolyP metabolism. Our findings establish that the DrPitA contributes to oxidative stress resistance of *D. radiodurans* by facilitating Mn-Pi enrichment.

**IMPORTANCE:**

As a model bacterium for extreme environmental adaptation, studies on the exceptional resistance of *Deinococcus radiodurans* have been focused on its DNA damage repair pathways and antioxidant systems. Among the antioxidant systems, the non-enzymic Mn-antioxidant complexes, including manganese-phosphate (Mn-Pi), which has efficient intracellular reactive oxygen species scavenging and protection of biomolecules from oxidative damage, play a significant role in stress resistance. However, the mechanism underlying Mn-Pi enrichment in *D. radiodurans* remains obscure. Therefore, investigating Mn-Pi transporter and their role in Mn-Pi homeostasis is important for understanding the oxidative stress resistance of this bacterium. In this study, we characterized the PitA homolog (DrPitA) as a transporter involved in Mn-Pi accumulation in *D. radiodurans*. We demonstrated that the DrPitA contributes to oxidative stress resistance through the acquisition of Mn ion and phosphate. These findings broaden understanding of the accumulation mechanism of non-enzymatic antioxidants in *D. radiodurans* and provide insights into how the extreme bacterium survives under oxidative stress.

## INTRODUCTION

*Deinococcus radiodurans* (DR), an extremophilic bacterium from the Deinococcus-Thermus phylum, possesses remarkable resistance to extreme stresses such as γ-ray irradiation up to 10 kGy. Its radiation tolerance is approximately 20-fold higher than that of *Escherichia coli* (EC) and 3,000-fold higher than that of human cells (1, 2). The radiation resistance of *D. radiodurans* was attributed to the efficient intracellular DNA damage repair system and strong antioxidant system (2–4). Ionizing radiation induces radiolysis of water molecules within cells, generating abundant reactive oxygen species (ROS) such as hydroxyl radicals (•OH) and superoxide anion radical (O_2_^−^•). These ROS inflict damage on intracellular proteins, nucleic acids, and other macromolecules, ultimately leading to cell death (1, 3, 5). Among the antioxidant systems of *D. radiodurans*, non-enzymatic antioxidants were considered crucial for cellular resistance to radiation-induced oxidative stress (6–8).

Radiation-resistant bacteria, including *D. radiodurans*, exhibit significantly higher intracellular concentrations of divalent manganese ions (Mn²^+^) and maintain high manganese/iron (Mn/Fe) ion ratios compared to radiation-sensitive bacteria (9, 10). A high Mn/Fe ion ratio is often indicative of a bacterium’s capacity to withstand severe oxidative stress (5, 11). This is primarily because elevated intracellular iron levels catalyze the production of highly damaging ROS, notably •OH, via the Fenton reaction (12, 13). In contrast, Mn²^+^ has a higher redox potential; thus, its chemical properties are relatively more stable (14). Mn²^+^ typically does not directly scavenge ROS; instead, it complexes with small molecule metabolites (e.g., orthophosphate, amino acids, peptides, and nucleotides) in a form as low-molecular-weight manganese complexes (LMW Mn-antioxidants or called small-molecule Mn antioxidants) exhibit catalytic ROS scavenging (2, 15, 16). These complexes effectively eliminate ROS, including O_2_^−^•, •OH, and hydrogen peroxide (H_2_O_2_) (8, 15). Analysis of ultrafiltrates (<3,500 Da) from bacteria such as DR, *Thermus thermophilus*, EC, and *Pseudomonas putida* revealed that the ultrafiltrate of *D. radiodurans* contained markedly higher concentrations of Mn²^+^, amino acids, nucleotides, and phosphates compared to radiation-sensitive bacteria, and these Mn-antioxidants present in the ultrafiltrate mitigated radiation-induced protein carbonylation damage at γ-radiation of doses up to 17.5 kGy (16). The Mn-antioxidants protect intracellular proteins from oxidative damage and play a pivotal role in bacterial oxidative stress defense. Crucially, manganese-phosphate complexes (Mn-Pi) found in radiation-resistant organisms like *D. radiodurans* can functionally substitute for superoxide dismutase (SOD) by scavenging O_2_^−^•. Supporting this, Mn-Pi complexes were shown to provide O_2_^−^• scavenging capability in MnSOD-deficient *Caenorhabditis elegans*, enabling its survival under extreme desiccation and oxidative stress conditions (15). However, the mechanism of Mn-Pi enrichment in *D. radiodurans* is still unclear.

Extracellular divalent metal ions (e.g., Mg^2+^, Ca^2+^, and Zn^2+^) and inorganic phosphate (Pi) can be co-transported into cells as metal-phosphate complexes (Me-Pi) by low-affinity inorganic phosphate transporters (PiT) utilizing the proton motive force (17, 18). For instance, *E. coli* PitA binds extracellular Mg²^+^ and Pi, facilitating their uptake as an Mg-Pi complex and maintaining intracellular metal and phosphorus homeostasis. Disruption of *pitA* significantly reduces intracellular Mg^2+^ levels and markedly slows the growth of *E. coli* (19, 20). In *Saccharomyces cerevisiae*, a functionally analogous low-affinity metal transporter Pho84 is involved in cellular antioxidant capacity by accumulating environmental Mn-Pi complexes (21). To date, whether PitA homologs are present and performing functions in the extremophilic *D. radiodurans* remains unknown. Moreover, we have previously identified that the inorganic phosphate polymer PolyP in *D. radiodurans* could complex with high concentrations of Mn^2+^, forming a PolyP-Mn complex (22). This complex can be degraded by exopolyphosphatase PPX to yield Mn-Pi complex under oxidative stress. The Mn-Pi demonstrates the ability to mitigate protein oxidative damage *in vitro*. Consistent with this finding, the *drppx* mutant exhibited impaired oxidative stress resistance (22).

In this study, we identify the role of the PitA homolog of *D. radiodurans* (DrPitA) for transporting and enriching Mn-Pi. We further analyzed the involvement of *drpitA* in Mn-Pi metabolism under oxidative stress, aiming to elucidate the mechanisms underlying Mn-Pi enrichment and its contribution to oxidative stress resistance of *D. radiodurans*.

## RESULTS

### Identification of PitA homolog in *D. radiodurans*

**Fig 1:**
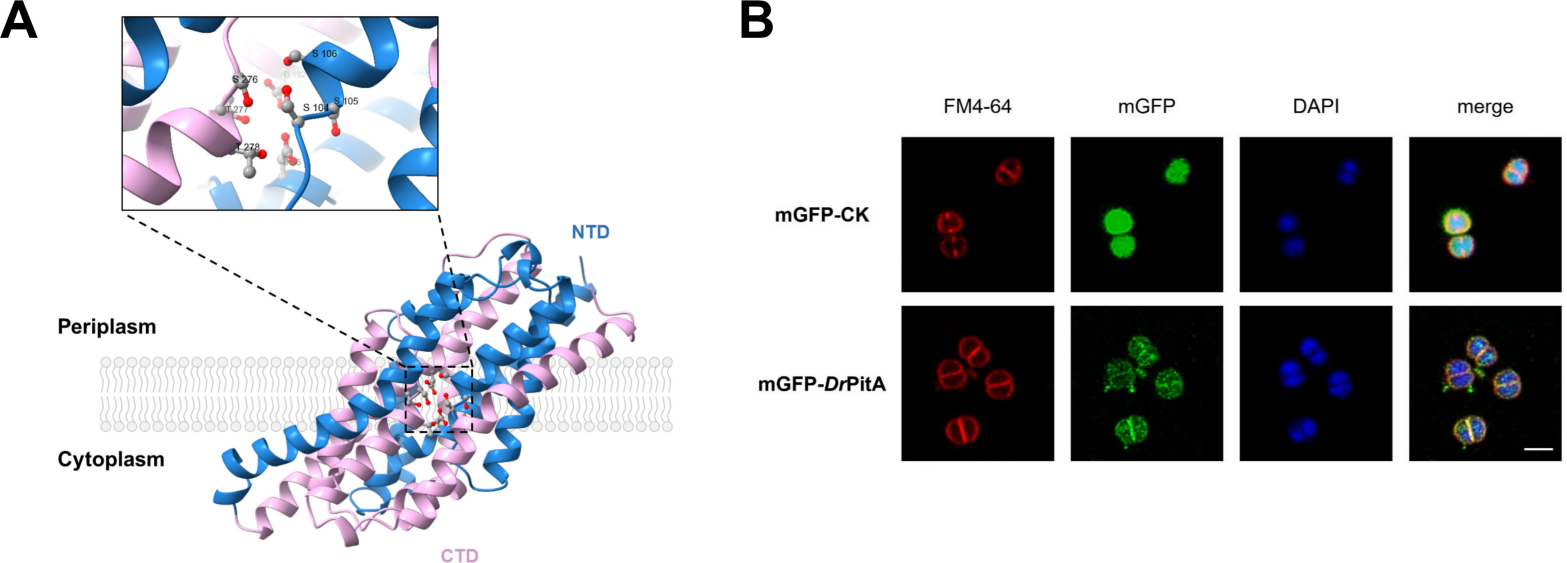
DrPitA comprises conserved domains and localizes to the cell membrane. (**A**) AlphaFold predicted structure of DrPitA. Enlarged regions show potential substrate (Mn-Pi) binding pockets. N-terminal and C-terminal are colored sky blue and light pink, respectively. (**B**) Subcellular localization of DrPitA. Top to bottom: mGFP-CK, control strain expressing mGFP; mGFP-DrPitA, mGFP-DrPitA fusion protein expressing strain. FM4-64, membrane staining; 4′,6-diamidino-2-phenylindole (DAPI), nucleoid staining; merge showing the co-localization of DrPitA-mGFP with membrane. Scale bar: 2 μm.

We queried the *D. radiodurans* genome with the PiT sequence from *Thermotoga maritima* to identify homologous proteins. This analysis revealed that the gene locus *dr0925* encodes a putative PitA homolog, sharing 30.2% sequence identity with the *T. maritima* PiT transporter, which belongs to the bacterial PHO4 superfamily comprising PiTs for metal-phosphate (Me-Pi) transport (23). The PitA homolog from *D. radiodurans* is designated DrPitA. Multiple sequence alignment of PitA homologs from *T. maritima*, *E. coli*, *Bacillus subtilis*, *Pseudomonas aeruginosa*, *Acinetobacter baumannii*, *Klebsiella pneumoniae*, and *D. radiodurans* identified conserved motifs located at the N-terminal (GXXDXAN and PXSXXH) and C-terminal (HGXND and PXSTTH) across the different species (Fig. S1), consistent with previous reports on PitA homologs (24). We next employed AlphaFold to predict the tertiary structure of DrPitA. The conserved motifs are likely involved in the substrate binding of DrPitA (Fig. 1A).

Transmembrane topology analysis of DrPitA predicted approximately 10 transmembrane helices interconnected by hydrophilic loops (Fig. S2A), suggesting that it is a transmembrane protein with a strong hydrophobic character (Fig. S2B). The predicted DrPitA structure also shows multiple transmembrane α-helices. Notably, conserved amino acid residues potentially forming the substrate binding pocket were localized within the core region of the structure (Fig. 1A). To experimentally determine the subcellular localization of DrPitA, we constructed a C-terminal mGFP fusion protein of DrPitA by cloning the *drpitA* gene fragment into the pRADK vector, generating pRADK-*drpitA*-GFP. A control plasmid expressing unfused mGFP (pRADK-GFP) was also generated. Both plasmids were transformed into wild-type R1 cells, and protein localization was assessed using confocal laser scanning microscopy. Fluorescence in the control strain (expressing mGFP) was uniformly distributed throughout the cytoplasm. In contrast, cells expressing the DrPitA-mGFP fusion exhibited a pronounced fluorescence signal specifically localized at the cell membrane (Fig. 1B), suggesting the DrPitA as a membrane protein.

**Fig 2:**
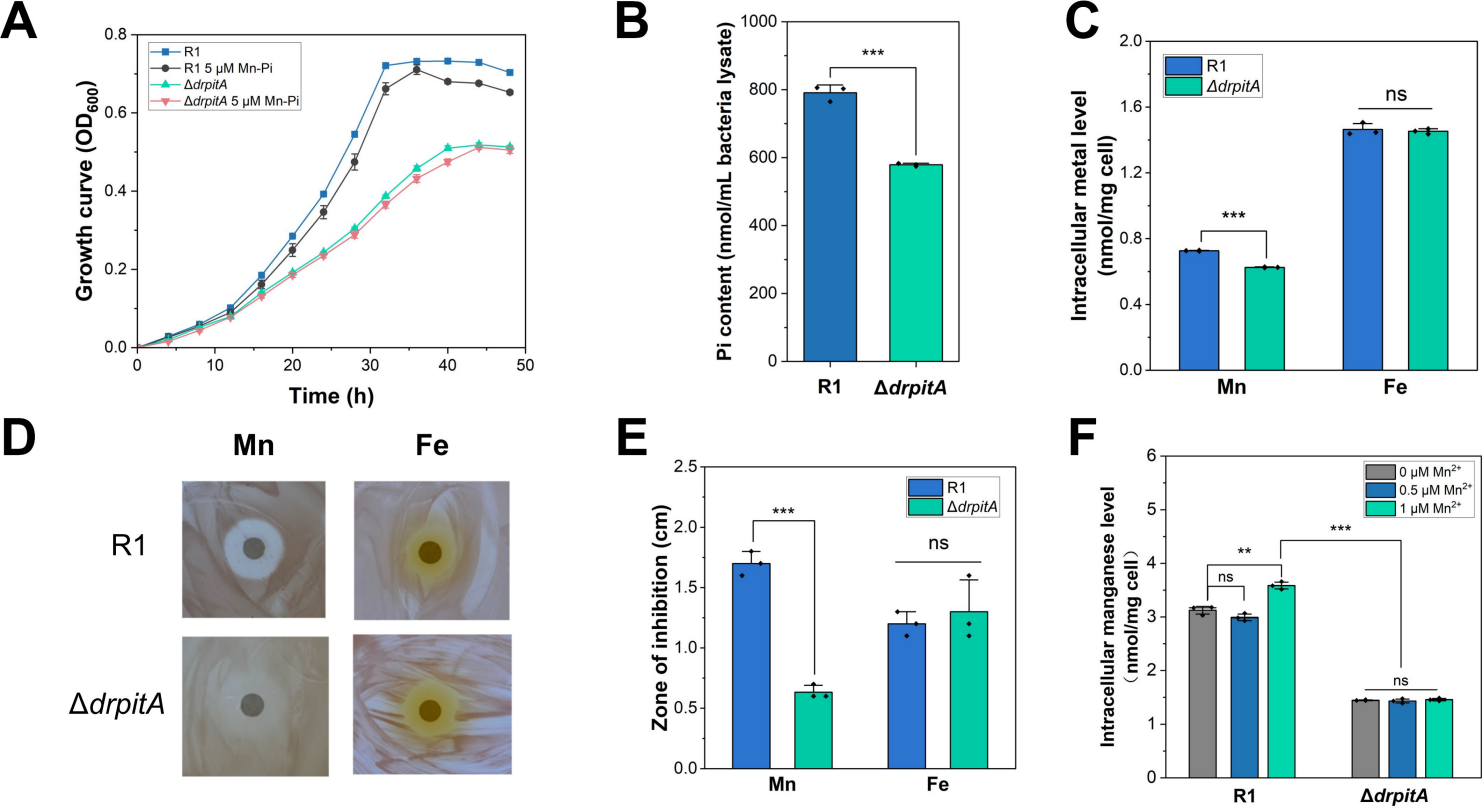
Mutation of *drpitA* impairs intracellular uptake of manganese and phosphate. (**A**) Growth curves of wild-type R1 and Δ*drpitA* mutant strains in restricted culture medium. (**B**) Intracellular free phosphate (Pi) levels in wild-type R1 and Δ*drpitA* mutants in restricted culture medium. (**C**) Intracellular metal ion content in wild-type R1 and Δ*drpitA* mutants grown in restrictive culture medium. (**D**) Inhibition zones following treatment with 1 M metal ions. (**E**) Quantification of inhibition zone diameters from panel D. (**F**) Effect of Mn ion supplementation in tryptone glucose yeast extract (TGY) medium on intracellular Mn levels in the wild-type R1 and Δ*drpitA* mutant. Data represent mean ± SD; ***, *P* < 0.001; **, *P* < 0.01; ns, not significant.

Bioinformatics and structural predictions imply that DrPitA likely participates in the co-transport of metal ion and phosphate (Me-Pi). To investigate the role of DrPitA in metal-phosphate transport, we constructed the DrPitA knockout mutation strain (Fig. S3). We compared the growth kinetics of wild-type R1 and Δ*drpitA* mutant strains in restricted culture medium. Growth curve analysis revealed that deletion of the *drpitA* gene delayed cell growth compared with the wild-type R1 under phosphate and manganese-limited conditions in restricted culture medium. Supplementing with 5 µM Mn-Pi complex failed to rescue the growth defect of the mutant (Fig. 2A). After gene complementation, the growth retardation caused by the *drpitA* gene deletion can be partially restored (Fig. S4). These results suggest that DrPitA might be important for the uptake and transport of Mn-Pi.

To assess the role of DrPitA in Mn-Pi accumulation, we quantified and compared intracellular phosphate and metal ion levels in the wild-type R1 and Δ*drpitA* strains cultured in the restricted culture medium. Using the molybdenum blue colorimetric method, we found that the wild-type cell lysate contained 0.8 μmol/mL of phosphate, while the phosphate content of the mutant strain was 0.6 μmol/mL. A significant reduction (27%, *P* < 0.001) of intracellular free phosphate content in the mutant strain compared with wild-type cells was shown (Fig. 2B). Furthermore, assays using inductively coupled plasma mass spectrometry (ICP-MS) showed that the wild-type cells contained 0.7 nmol of Mn per milligram of dry cells, while the Mn content in the mutant strain was 0.6 nmol/mg of dry cells, representing a 14.3% decrease compared to the wild type. In contrast, iron (Fe) content in the Δ*drpitA* mutant remained similar to that of the wild type (Fig. 2C). These findings indicate that DrPitA is involved in intracellular Mn-Pi accumulation in *D. radiodurans* by facilitating the uptake of phosphate and Mn ions.

Lack of key transport proteins can impair ion uptake and enrichment and potentially reduce cellular sensitivity to specific ions. Inhibition zone assays are commonly employed to assess the impact of gene mutations on metal ion sensitivity and validate the function of related transporter. The inhibition zone of metal ion assays revealed differences in metal sensitivity between the wild-type R1 and Δ*drpitA* mutant strains. Under treatment with 1 M Mn^2+^, the mutant consistently exhibited significantly smaller inhibition zones compared to the wild type, where the mutant displayed a marked decrease in Mn ion sensitivity (*P* < 0.001; Fig. 2D and E). In contrast, no significant difference in inhibition zone was detected between the mutant and wild type following Fe^2+^ treatment (Fig. 2E), indicating that DrPitA is not involved in Fe^2+^ transport. The reduced inhibition zones observed for Mn^2+^ are likely attributable to impaired uptake and enrichment of Mn^2+^ in the Δ*drpitA* mutant. Collectively, the metal ion sensitivity demonstrates that DrPitA participates in Mn ion transport and is crucial for maintaining Mn ion homeostasis in *D. radiodurans*.

Next, we supplemented TGY medium with varying concentrations of MnCl_2_ (0.5 µM and 1 µM) and measured intracellular manganese levels in wild-type R1 and Δ*drpitA* mutant strains using ICP-MS. Following supplementation with 1 µM MnCl₂, the intracellular Mn content in the wild-type R1 cells was approximately 3.6 nmol/mg of dry cells, while the Δ*drpitA* mutant strain contained approximately 1.5 nmol/mg of dry cells. The intracellular manganese content of the wild-type cells was more than twice that of the Δ*drpitA* mutant under supplementation with 1 µM MnCl_2_ (Fig. 2F). Compared to wild-type cells grown in TGY medium without additional Mn ions, the Mn content in R1 grown with supplemented 1 µM MnCl₂ increased by 14.7% (*P* < 0.01). In contrast, the manganese content in the Δ*drpitA* mutant showed no significant change upon MnCl_2_ addition at either concentration (Fig. 2F, *P* > 0.05). These results support that DrPitA plays a critical role in Mn ion uptake and accumulation in *D. radiodurans*.

### Mutation of *drpitA* leads to a decrease in the oxidative stress-resistance of *D. radiodurans*

**Fig 3:**
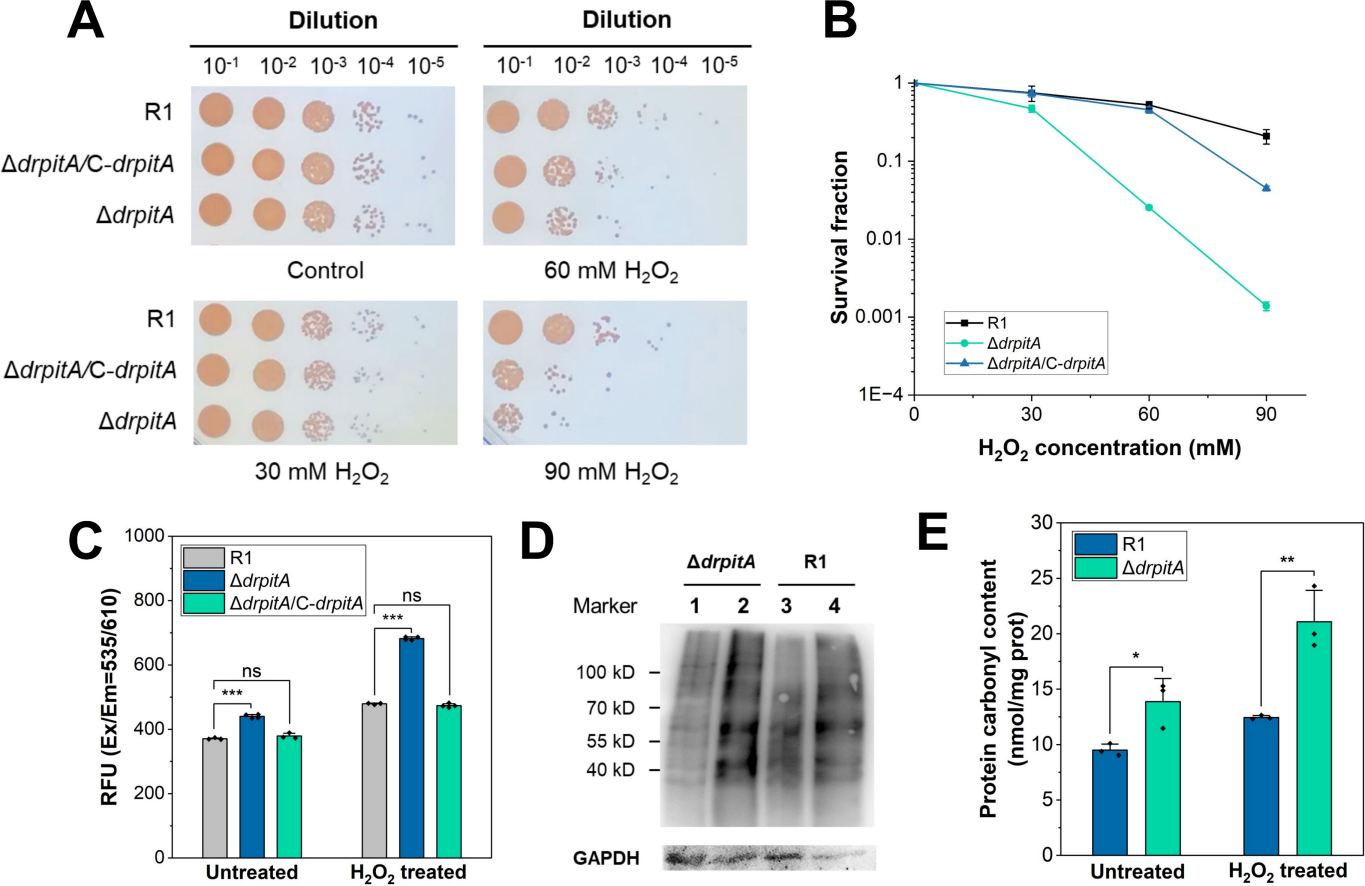
DrPitA contributes to oxidative stress resistance of *D. radiodurans*. (**A**) Survival phenotypes of wild-type R1, Δ*drpitA*, and complemented strain (Δ*drpitA*/C-*drpitA*) after treatment with increasing concentrations of H_2_O_2_ stress for 30 min. Control, cells in the absence of H_2_O_2_ treatment. (**B**) Survival curves after H_2_O_2_ exposure. Mid-log phase cells (OD_600_ = 1.0; ~10^7^ CFU/ml in Tris-HCl pH 7.0) were treated with H_2_O_2_ for 30 min. Viability was determined by colony counts on TGY plates after 3-day incubation. (**C**) Intracellular ROS levels in the wild-type R1, Δ*drpitA*, and Δ*drpitA*/C-*drpitA* strains after 30 min H_2_O_2_ treatment. (**D**) Western blot analysis of protein carbonylation in cell lysates. Lanes: 1: Δ*drpitA* (untreated), 2: Δ*drpitA* + H_2_O_2_, 3: R1 (untreated), 4: R1 + H_2_O_2_. GAPDH was used as a reference protein. (**E**) Quantification of protein carbonyl levels using spectrophotometry. Data represent mean ± SD (*n* = 3); ***, *P* < 0.001; **, *P* < 0.01; *, *P* < 0.05; ns, not significant.

We compared the survival phenotypes and fractions of wild-type R1, Δ*drpitA* mutant, and complemented strain (Δ*drpitA*/C-*drpitA*) under H_2_O_2_ stress. Results showed significantly reduced survival in the mutant (Fig. 3A), with a survival fraction dropping to 4.8% of the wild type at 60 mM H_2_O_2_, and it plummeted to 0.7% of the wild type at 90 mM H_2_O_2_ (Fig. 3B). Gene complementation of the Δ*drpitA* could restore the survival fraction close to the level of the wild-type strain (Fig. 3B), indicating the contribution of DrPitA in oxidative stress resistance of *D. radiodurans*.

To elucidate mechanisms underlying the survival defect of the *ΔdrpitA* under oxidative stress, we analyzed the intracellular ROS levels in wild-type R1, Δ*drpitA*, and complemented strain (Δ*drpitA*/C-*drpitA*) following H_2_O_2_ exposure. Following treatment with 30 mM H_2_O_2_ for 30 min, all the strains showed elevated ROS accumulation, with the Δ*drpitA* mutant exhibiting markedly higher ROS levels (1.4-fold of the wild type, *P* < 0.001) than both the wild-type and the complemented strain (Fig. 3C). These findings imply that DrPitA was involved in reducing intracellular ROS through its transport and enrichment of Mn-Pi antioxidant. Protein carbonylation serves as a key biomarker of protein oxidative damage. Western blot analysis revealed an elevated protein carbonylation in Δ*drpitA* versus the wild-type R1 (Fig. 3D). A spectrophotometric assay with a DNPH probe revealed that the *drpitA* mutant suffered severe H_2_O_2_-induced protein damage. After 30 min of hydrogen peroxide treatment, approximately 21.1 nmol carbonyls per milligram of protein was detected for the mutant, which was nearly 1.7-fold of the wild-type cells (Fig. 3E). This extensive protein oxidation explains the decrease in the mutant’s survival under oxidative stress.

### DrPitA contributes to oxidative stress resistance through participating in Mn and phosphate metabolism

**Fig 4:**
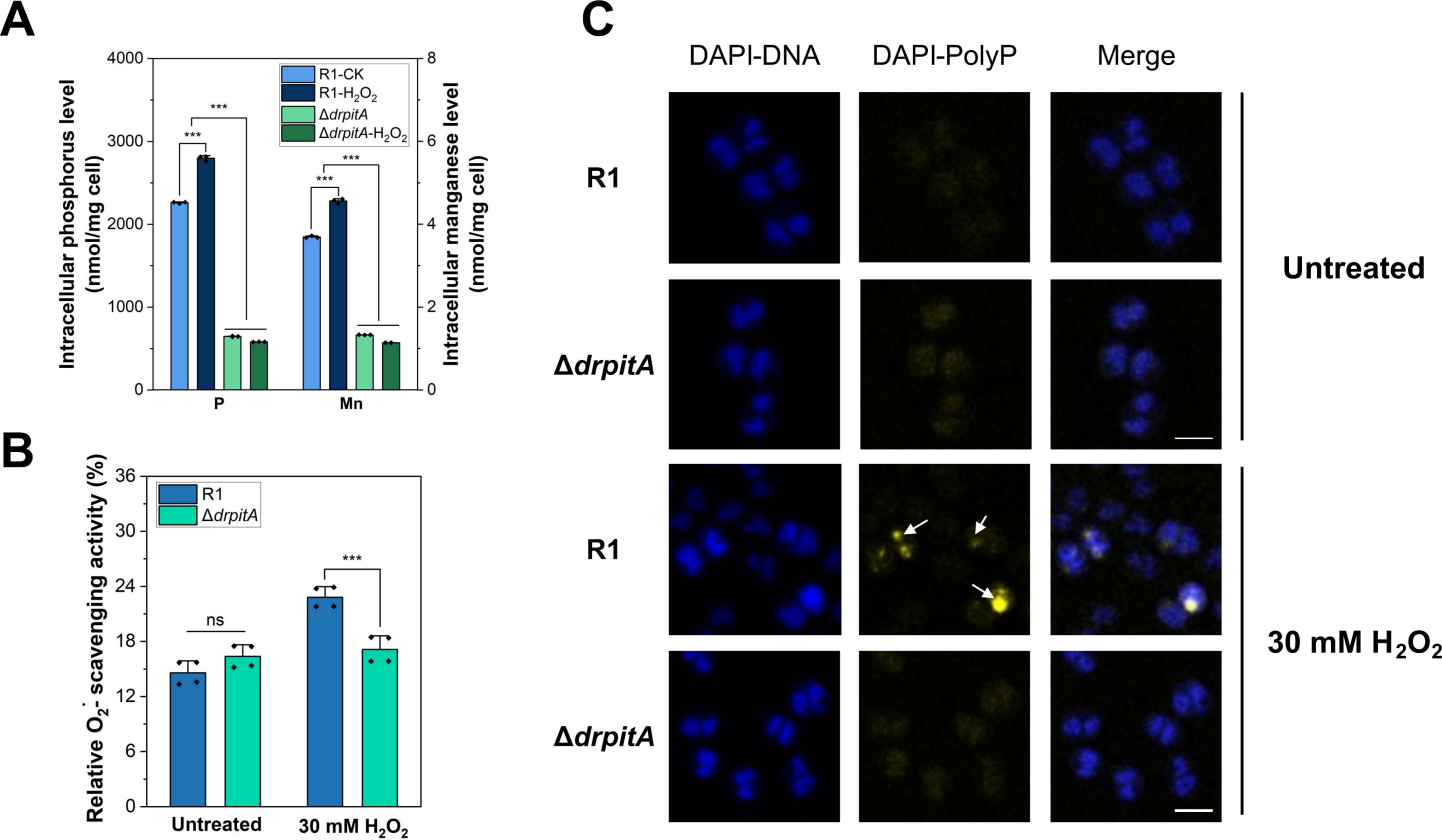
DrPitA is involved in oxidative stress resistance by increasing the accumulation of intracellular Mn and P. (**A**) Intracellular phosphorus and manganese content in wild-type R1 and Δ*drpitA* grown in TGY medium with or without H_2_O_2_ treatment. (**B**) Superoxide radical (O_2_^−^•) scavenging capacity of cell lysates from the wild-type R1 and Δ*drpitA*. (**C**) Confocal laser scanning microscopy imaging of intracellular PolyP in wild-type R1 and Δ*drpitA* before and after 30 mM H_2_O_2_ treatment. White arrows indicate PolyP (yellow fluorescence). Scale bar: 2 μm. Data represent mean ± SD; ***, *P* < 0.001; ns, not significant.

Real-time quantitative PCR (qRT-PCR) analysis of *drpitA* transcription under 40 mM H_2_O_2_ stress showed a time-dependent upregulation in the wild-type R1 (0–60 min), peaking with significant induction at 15 min after stress with an mRNA level increasing (1.4 times of the control, *P* < 0.01), and subsequently declined (Fig. S5), consistent with a stress response and post-stress recovery. Analysis of cellular Mn/P content in the wild-type R1 and Δ*drpitA* pre- and post-H_2_O_2_ exposure revealed that the wild-type R1 exhibited an elevation of Mn/P level post-H_2_O_2_ exposure (*P* < 0.001). After treatment with hydrogen peroxide, the phosphorus content of wild-type increased from 2.3 μmol/mg to 2.8 μmol/mg of dry cells, while the manganese content rose from 3.7 nmol/mg to 4.6 nmol/mg of dry cells (Fig. 4A). Whereas the Δ*drpitA* showed no increase in Mn/P level post-H_2_O_2_ stress. The Mn/P levels in the mutant were lower than those of the wild-type counterparts following H_2_O_2_ treatment. Furthermore, we assayed the O_2_^−^• scavenging capacity in cell lysates (Fig. 4B). The cell lysates from wild-type R1 post-H_2_O_2_ induction exhibited 1.6-fold O_2_^−^• scavenging activity compared with the untreated wild-type R1 and significantly higher than the O_2_^−^• scavenging activity of the Δ*drpitA* mutant, indicating that oxidative stress-induced accumulation of Mn-Pi antioxidant with the assistance of DrPitA enhanced the ROS scavenging capacity of *D. radiodurans* wild-type cells.

Given that *drpitA* mutation disrupts intracellular phosphate homeostasis, we hypothesized that there would be concomitant transcription changes of phosphate storage-related genes. Our previous study has shown that free Mn ions can be chelated by intracellular polyphosphate (PolyP) to form a PolyP-Mn pool, which serves as a storage for Mn and phosphate within the cell of *D. radiodurans* (22). In *D. radiodurans*, DrPPK synthesizes PolyP, while DrPPX hydrolyzes PolyP to liberate phosphate (22). qRT-PCR analysis revealed elevated transcription levels of both *drppk* (1.3-fold) and *drppx* (1.7-fold) in the Δ*drpitA* mutant compared to the wild-type R1 (Fig. S6A). This upregulation likely represents a compensatory cellular response to *drpitA* deficiency. Using confocal microscopy analysis, we investigated the PolyP levels in Δ*drpitA* mutant and the wild-type strain. The wild-type cells exhibited a PolyP accumulation under H_2_O_2_ stress, while the Δ*drpitA* mutant had no substantial PolyP accumulation (Fig. 4C). The fluorescence quantification of PolyP also showed that the relative PolyP level in the wild type after hydrogen peroxide treatment was approximately 1.7 times that of the untreated wild type; however, the relative PolyP level in the Δ*drpitA* mutant strain showed non-significant change (Fig. S7). Previously established PolyP-Mn dynamics proposed that oxidative stress triggers the DrPPX-mediated hydrolysis of the PolyP-Mn to release Mn-Pi antioxidants (3, 22). The deficiency of DrPitA may result in the limiting formation of intracellular PolyP-Mn pools through a decrease of Mn-Pi uptake; consequently, the impaired PolyP-Mn storage reduces bioavailable Mn-Pi for ROS scavenging. Therefore, DrPitA may be related to the regulation of PolyP metabolic homeostasis.

## DISCUSSION

This study demonstrates that DrPitA mediates Mn-Pi accumulation and PolyP metabolic modulation, which contributes to oxidative stress resistance of *D. radiodurans*. Evidence from several lines supports the results. First, the *drpitA* mutant strain showed greater sensitivity to oxidative stress, and the intracellular ROS levels of the mutant strain were significantly higher than wild type under oxidative stress. Second, the transcription level of the *drpitA* gene was increased, and intracellular Mn/P accumulation in the wild-type strains was significantly increased under oxidative stress compared with the mutant strain. In addition, the deletion of *drpitA* resulted in a relatively lower intracellular PolyP level in the mutant strain compared to the wild-type strain.

Fluorescence localization confirmed the membrane location of DrPitA. The *drpitA* mutant exhibited significantly depleted Mn and Pi, indicating DrPitA mediates cotransport of Mn^2+^ and Pi to maintain cellular homeostasis of Mn-Pi. While PitA homologs in *E. coli*, *Acinetobacter johnsonii*, and *Bacillus subtilis* show broad divalent metal specificity (Mg^2+^/Ca^2+^/Zn^2+^/Co^2+^) (25, 26), the DrPitA exhibits a possible preference for Mn²^+^ specificity. The intracellular copper and zinc content in the wild-type and Δ*drpitA* mutant strains was measured. There was little difference in copper content between the wild-type and Δ*drpitA* mutant, while a very slight increase in zinc content was observed in the Δ*drpitA* mutant (Fig. S8). Yin et al. demonstrated that *E. coli* MgtS forms binary complexes with PitA to prevent Mg^2+^ efflux, with sRNA MgtR suppressing the *pitA* transcription (20). The transport mechanism and metal ion preference of DrPitA need further investigation using protein structure analysis.

Under oxidative stress, the transcription level of the *drpitA* gene in wild strains was significantly upregulated, indicating a response to oxidative stress. Culotta and Daly found that oxidative stress can induce Mn-Pi transporters in eukaryotic yeast cells, which may be controlled by the Pho80p/Pho85p signaling pathway (8, 21). Chrystala et al*.* found that the *pitA* homologous genes in *E. coli* may be regulated by the CRP/FNR (cAMP receptor protein/fumarate and nitrate reduction) family transcription regulatory factors (27). Further in-depth research is needed on the regulatory pathways and mechanisms of the *drpitA* gene. In addition, oxidative stress may also induce other oxidative stress-response systems in *D. radiodurans*, such as PprI-DdrO and quorum-sensing DqsIR found in previous studies (28, 29), which may jointly participate in the stress response.

Bacterial intracellular ion homeostasis might be maintained through coordinated or compensatory mechanisms involving multiple transport systems. In *D. radiodurans*, Mn ion uptake is facilitated by additional systems, including the MntH encoded by *dr1709* (30) and the MntABC transporter homologs encoded by *dr2283*, *dr2284*, and *dr2523* (31). The DtxR-family transcriptional repressor (DR2539) was proposed to negatively regulate *mntH* expression, while FeoB (DR1219) might mediate iron ion transport (25, 26). To evaluate the transcriptional impact of *drpitA* deletion on the other manganese homeostasis-related genes, we performed qRT-PCR analysis (Fig. S6B). Deletion of *drpitA* triggered compensatory upregulation of manganese homeostasis genes, such as *mntABC* transporters, and a reduction in transcript levels of the repressor gene *dtxR* in the mutant. The downregulation of *dtxR* likely derepressed its target manganese transporter genes, leading to elevated transcription of *mntH*, *mntA*, and *mntB*. These results likely explain the observed residual intracellular manganese levels in the Δ*drpitA* mutant. Intracellular PolyP serves as a dynamic Mn-Pi reservoir. PolyP molecules inside *D. radiodurans* can form PolyP-Mn complex with high concentrations of Mn ions, and Mn-Pi can be released from PolyP-Mn complex by DrPPX (22). The knockout of the *drpitA* gene under oxidative stress resulted in a lower accumulation level of intracellular PolyP compared to the wild-type strain, accompanied by a decrease in resistance to oxidative stress. This may be due to the relatively lower levels of Mn ions and phosphate substrates in the *drpitA* mutant strain compared to the wild-type strain. Therefore, DrPitA may participate in Mn-Pi transport and impact on PolyP metabolic pathways. Further studies on the dynamic changes of intracellular PolyP-Mn and Mn-Pi are needed through a combination of gene knockout, combined with higher resolution *in situ* analysis techniques. A recently published paper reported that some ROS assays using dihydrodichlorofluorescein derivatives may not be valid to reflect the intracellular ROS level in bacteria (32). In our study, we employed an alternative and more suitable dihydroethidium (DHE) as the probe for ROS level analysis, and a protein carbonylation detection assay was also used to jointly corroborate our results.

In summary, our results established that DrPitA contributes to oxidative stress resistance of *D. radiodurans* by facilitating Mn-Pi enrichment. These findings broaden our understanding of the accumulation mechanism of non-enzymatic antioxidants in *D. radiodurans* and provide insights into its resistance to extreme oxidative stress.

## MATERIALS AND METHODS

### Bacterial strains and plasmids

The strains and plasmids involved in this study are shown in Table 1. *Deinococcus radiodurans* R1 (ATCC13939) was cultured in TGY medium (0.5% tryptone, 0.1% glucose, 0.3% yeast extract, and solid medium with 1.5% agar powder) at a temperature of 30°C. *E. coli* was cultured on lysogeny broth (LB) medium (1% yeast extract, 0.5% tryptone, 1% sodium chloride, and solid medium with 1.5% agar powder) at a temperature of 37°C. The concentration of kanamycin sulfate (Kan) used for *D. radiodurans* screening is 35 µg/mL, and the concentration of chloramphenicol is 5 µg/mL. For screening *E. coli*, the concentration of kanamycin sulfate used is 50 µg/mL, the tetracycline (Tet) concentration is 12.5 µg/mL, and the ampicillin (Amp) concentration is 100 µg/mL. Primers used are shown in Table 2 and Table S1, and the primer synthesis was provided by Shanghai Jierui Bioengineering Co., Ltd. The DNA sequencing was completed by Hangzhou Qingke Biotechnology Co., Ltd.

**TABLE 1:** Strains and plasmids used in this study

Strain or plasmid	Description	Source
Strains		
*D*. *radiodurans* R1	Wild-type strain, ATCC 13939	Lab stock
*ΔdrpitA*	Knockout mutants of R1 deleted *pitA*, Kan^R^	This study
*ΔdrpitA/*C*-drpitA*	*ΔdrpitA* complemented with pRAD-*drpitA*	This study
*E. coli* DH5α	F- φ80*lacZΔM15 Δ(lacZYA-argF) U169 recA1 endA1 hsdR17(rk- mk+) phoA supE44 thi-1 gyrA96 relA*1 λ-	ToloBio
Plasmids		
pRADK	*E. coli-D. radiodurans* shuttle vector carrying *D. radiodurans* groEL promoter (Tet^r^ kan^r^ Cm^r^)	Lab stock
pRAD-*drpitA*	pRADK derivative in which the kanamycin gene is replaced with *drpitA*	This study
pRAD-*drpitA-gfp*	pRADK derivative in which the kanamycin gene is replaced with *drpitA* and *gfp*	This study
pRAD-*gfp*	pRADK derivative in which the kanamycin gene is replaced with *gfp*	This study
pUC19	For DNA cloning (Amp^r^)	Lab stock
pUC19-*ΔdrpitA*	pUC19 ligated with DNA fragments used for *drpitA* mutant construction	This study

**TABLE 2:** Primers used in this study

Primer name	Sequence (5′−3′)^*a*^
Construction of *drpitA* mutant	
*drpitA*-P1	ACCATGATTACGCCAAGCTTACCACGGTGTCACTGCTGCGGG
*drpitA*-P2	TGCTCGATGAGTTTTTCTAGGGATCCGCTGGCTCTGGTCTCCTCTGTCCCT
*drpitA*-P3	GGCGACAATACGTGCTTCCAAAGCTTGCGCCTAGCAAGCAGAAAGAGAGGC
*drpitA*-P4	AGCTCGGTACCCGGGGATCCCACTGGGCCTGAGCGAAACAGTGCT
*drpitA*-P5	TTCGACTTCATCAACGGCTTTCACG
*drpitA*-P6	CAGGCGTAGATCACCCAGTGAGGAA
Kana-F	AAGCTTTGGAAGCACGTATTGT
Kana-R	CTAGAAAAACTCATCGAGCA
pUC19-F	GGATCCCCGGGTACCGAGCTC
pUC19-R	AAGCTTGGCGTAATCATGGTCATAGCTG
Compensatory plasmids	
pRAD-F	AGCAGCGGCCTGGTGCCGCGCGGCAGC
pRAD-R	TGATAGATACAAAGAACACGTCAAG
*drpitA*-F	AGCGGCCTGGTGCCGCGCGGCAGCCATATGGAAACTGCACTGATCGTCCTGAT
*drpitA*-R	GGAGCTCGAATTCGGATCCTCAGTTCCCGCCCGCCAG
Construction of GFP-fused DrPitA	
rec-HRV3C-0925-R	CAGCACTTCCAGACCACCAGAACCACCAGAACCACCGTTCCCGCCCGCCAGCAAG
rec-HRV3C-mGFP-F	TCTGGTGGTCTGGAAGTGCTGTTTCAGGGTCCGCATATGGTTTCCAAAGGCGAAG
rec-mGFP-F	CTGGTGCCGCGCGGCAGCCATATGGTTTCCAAAGGCGAAGAACTGT
rec-mGFP-R	CGGAGCTCGAATTCGGATCCTCATTTGTACAGCTCGTCCATACCCAGG

^*a*^ Underlined sequences indicate a homologous DNA fragment.

### Bioinformatics analysis

The related gene and protein sequences are obtained from the KEGG gene database (https://www.kegg.jp/) and UniProt protein data database (https://www.uniprot.org/). PitA homologous protein sequence was screened using Protein BLAST tool in NCBI database (https://blast.ncbi.nlm.nih.gov/Blast.cgi). *Thermotoga maritima* MSB8 PiT protein sequence (KEGG number: TM0261) was used as the reference sequence. The ClustalW software was used for homologous protein sequence alignment. The homologous protein sequence alignment results were processed using the online processing tool ESPrip (https://espript.ibcp.fr/ESPript/cgi-bin/ESPript.cgi). Online analysis tool TMHMM-2.0 was used for predicting protein transmembrane regions (https://services.healthtech.dtu.dk/services/TMHMM-2.0/). Protein hydrophilicity and hydrophobicity were analyzed using ProtScale (https://web.expasy.org/protscale/). The AlphaFold database (https://alphafold.ebi.ac.uk/) was used for protein structure prediction analysis, and the relevant protein structures were processed and presented using Pymol software (v2.4.1).

### Construction of *drpitA* knockout strain and complemented strain

The construction of gene knockout strains adopts the homologous recombination method, using DNA fragments of about 1,000 bp upstream and downstream of the target gene as homologous arms, and utilizing the homologous recombination ability of *D. radiodurans* to replace the target gene fragment with a resistance gene fragment (Fig. S3). The target gene knockout strain was obtained by screening with appropriate concentrations of antibiotics. Primers used for the construction of *drpitA* knockout strain and the complemented strain are listed in Table 2.

The plasmid pUC19-Δ*drpitA* containing the *drpitA* knockout DNA fragment was screened, and two primers, *drpitA*-P1/P4, were designed to clone the DNA fragment containing the kana resistance gene. The *drpitA* gene knockout strain was validated using *drpitA*-P5/P6 and Kana-F/R primers and DNA sequencing.

For complemented strain construction, the *drpitA* gene fragments were recombined onto the pRADK plasmid using a homologous recombination kit (2× MultiF Seamless Assembly Mix, ABconal). The recombinant product was transformed into *E. coli* DH5α competent cells, screened using LB agar plates containing tetracycline resistance, cultured at 37°C until the colonies were visible, and selected colonies for sequencing. The pRAD-*drpitA* plasmid was transformed into the above-mentioned gene knockout strain. Using a TGY agar plate containing 5 µg/mL chloramphenicol and 35 µg/mL kanamycin sulfate, the *drpitA* complemented strain was screened and obtained.

### Cellular localization of DrPitA

In order to investigate the distribution and localization of DrPitA protein in cells, the *drpitA* gene was fused with enhanced green fluorescent protein (mGFP, Ex/Em = 488/510 nm) gene in pRADK shuttle plasmid to obtain the pRAD-*drpitA-gfp* and pRAD-*gfp* plasmid as the method described previously (33). The recombinant plasmid was transformed into wild-type R1 competent cells and screened using TGY agar plates with 5 µg/mL chloramphenicol. Colony was selected for sequencing validation. After being cultured in 5 mL of TGY liquid medium containing chloramphenicol at 30°C until the exponential growth stage (OD_600_ = 0.8–1.0), 1 mL of the bacterial culture was centrifuged at 3,000 *× g* for 5 min, and the precipitate was washed twice with PBS buffer. The cells were added with 1 μL of 10× FM4-64 cell membrane red fluorescent dye (Sangon, Ex/Em = 510/750 nm) and 1 μL of 10× 4′,6-diamidino-2-phenylindole (DAPI) DNA fluorescent dye (Sangon, Ex/Em = 405/460 nm). Bacterial cells were observed using a confocal laser scanning microscope (Leica TCS SP8 STED3X) and analyzed using LAS X (v3.3.0) software.

### Growth curve

The wild-type R1, knockout strain, and complemented strain were cultured in 5 mL 3-(N-morpholino) propanesulfonic acid (MOPS)-restricted culture medium or TGY medium. The main components of MOPS restricted culture medium are MOPS (40 mM), K_2_HPO_4_ (0.132 M), AUGC bases (2 mM, dissolved in 0.015 M KOH), 20 essential amino acids (0.1 mM), 0.1% glucose, vitamin B, and trace metal elements (containing 5 μM MnCl_2_). The configuration method of Mn-Pi refers to the method of Daly et al. (16): mixing MnCl_2_ solution with pH 7.0 phosphate buffer solution and adjusting the final concentration of Mn^2+^ to 5 μM. The OD_600_ value was recorded every 4 h using a spectrophotometer.

### Intracellular phosphate content analysis using the molybdenum blue colorimetric method

The detection for intracellular phosphate was referring to the molybdenum blue colorimetric method as described by Holman et al. (34). The absorbance value of the generated molybdenum blue at 660 nm is proportional to the concentration of phosphate (Pi) in the solution. KH_2_PO_4_ standard solutions (25, 50, 100, 250, and 500 nmol) were prepared for standard curves. Take 50 mL of wild-type R1 and knockout strain (OD_600_ = 1.0) cultures, centrifuge to remove the supernatant, and wash the bacterial pellets once with sterile ddH_2_O. The precipitate was thoroughly suspended in 5 mL of lysis buffer (20 mM Tris HCl pH 8.0, 500 mM NaCl) and sonicated at 650 W power for 1 h. The lysate was centrifuged at 12,000 rpm for 20 min, and the supernatant was collected. A total of 500 μL of the supernatant was added with 200 μL of ammonium molybdate solution (50 g/L, dissolved in 2 M H_2_SO_4_), then 100 μL of sodium sulfite solution (200 g/L) and 100 μL of hydroquinone solution (5 g/L) were added to react at room temperature for 30 min. After the reaction, 200 μL of the reaction solution was used to measure the absorbance at 660 nm (A_660_).

### Metal cation sensitivity assessment

The metal ion sensitivity assay is commonly used to evaluate the phenotype of bacterial cells under metal ion stress and characterize the corresponding metal transporter function (30). A total of 100 μL of wild-type R1 and *drpitA* gene knockout strain (OD_600_ = 1.0) was taken and evenly coated onto TGY agar plates and dried. A 1 M MnCl_2_ or FeCl_2_ solution was prepared and filtered using a 0.22 μm filtration membrane for later use. Sterilized filter paper (with a diameter of about 5 mm) was spread flat on a TGY agar plate coated with bacteria. Five microliters of metal ion solution was added to the center of the filter paper. The plates were placed in a 30°C incubator and cultured for 48 h. The distance from the edge of the colony to the center of the filter paper was measured.

### RNA extraction and real-time quantitative PCR analysis

qRT-PCR was used to test the transcriptional differences of manganese and phosphate homeostasis-related genes in wild-type R1 and *drpitA* knockout strains. One milliliter of freshly cultured wild-type R1 and knockout strain bacterial fluid (OD_600_ = 1.0) was centrifuged at 12,000 × *g* for 1 min to collect the bacterial cells, resuspended in 200 μL of lysozyme solution dissolved in diethyl pyrocarbonate (DEPC)-treated water, and incubated at 37°C for 1 h to fully lyse the cells. Total RNA was extracted using TransZol Up Plus RNA Kit (TransGen) for extraction. Preparation of the cDNA Library was performed using the GoScript Reverse Transcriptase kit (Promega). qRT-PCR primers were as listed in Table S1. *dr1343* (encoding 3-phosphoglyceraldehyde dehydrogenase GAPDH) was used as an internal reference gene. qRT-PCR was performed using the TB Green Premix Ex Taq II (Takara) kit on an Agilent Stratagene Mx3005P instrument.

### Survival assay under oxidative stress

Survival phenotype and fractions of bacterial cells under oxidative stress were analyzed using the method as described previously (22). For the survival phenotype, the wild-type R1, *drpitA* mutant, and complemented strains were cultured until OD_600_ = 1.0. The bacterial culture was centrifuged at 3,000 *× g* for 5 min, and the precipitate was washed twice with 20 mM Tris HCl pH 7.0. The bacterial pellet was suspended in Tris-HCl buffer and was added with H_2_O_2_ solution at different final concentrations (0, 30, 60, and 90 mM). The mixture was incubated at room temperature for 30 min. After treatment, the bacterial cells were centrifuged at 3,000 *× g* for 5 min to remove the supernatant. The precipitate was fully suspended in 1 mL Tris-HCl buffer. A total of 100 μL of the suspension was added to 900 μL Tris-HCl buffer and mixed well. The mixture was then diluted in a 10-fold gradient until it reached a dilution of 10^−5^. A total of 5 μL suspension with different dilutions was transferred to stress-free TGY agar plates, incubated at 30°C for 48 h, and the formation of bacterial colonies was monitored.

For the survival fraction assays, the bacterial cells at OD_600_ = 1.0 were subjected to 30 min of stress treatment using different concentrations of hydrogen peroxide. After treatment, centrifuge to remove the supernatant and suspend the precipitate in 1 mL Tris-HCl buffer. A total of 100 μL of bacterial cell suspension with an appropriate dilution was coated onto stress-free TGY plates. After 48 h of cultivation in a 30°C incubator, the colony counting was performed, and the survival curve was plotted based on the colony-forming unit.

### Determination of intracellular ROS level

Intracellular ROS level was measured using the DHE probe (Solarbio, China). DHE is one of the most commonly used membrane-permeable fluorescence detection probes for superoxide anion. DHE can combine with protein to produce blue fluorescence after entering living cells. When oxidized by superoxide anion, the oxidation product ethidium can combine with DNA to produce red fluorescence, which can represent the relative content of intracellular ROS. Briefly, 1 mL of bacterial culture (OD_600_ = 1.0) was centrifuged at 3,000 *× g* for 5 min to remove the supernatant and wash the precipitate once with 20 mM Tris-HCl pH 7.0. The bacterial cells were divided into two equal parts; one was used as a control, and the other group was incubated for 30 min in Tris-HCl buffer containing 30 mM hydrogen peroxide. After the incubation, the cells were washed with 1 mL Tris-HCl buffer and incubated with DHE at a final concentration of 100 μM for 30 min at 37°C. Fluorescence intensity of the bacterial cells was measured using SpectraMax M5 Microplate Reader (Molecular Devices). Excitation and emission wavelengths were set at 535 nm and 610 nm, respectively.

### Protein carbonylation analysis

Proteins are oxidized (carbonylated) when exposed to intracellular ROS. 2,4-Dinitrophenylhydrazine (DNPH) derivatizes the carbonyl group to form 2,4-dinitrophenylhydrazone (DNP hydrazone). DNP hydrazone has a characteristic absorption peak at 370 nm. A total of 160 μL of bacterial lysate containing equal amounts of protein was taken for protein carbonylation analysis according to the instructions of the kit (Solarbio, China). The absorbance value of the DNP derivatization was measured at 370 nm, and the carbonyl level was calculated based on the protein concentration as nanomoles per milligram protein.

Western blot detection of protein carbonylation was performed using the Protein Carbonylation Detection Kit (ab178020, Abcam, China). The samples were transferred to the polyvinylidene fluoride (PVDF) membrane and incubated with anti-DNP primary antibody or anti-GAPDH primary antibody. After incubating at 4°C for 16 h, HRP-conjugated secondary antibody was added and incubated at room temperature for 1 h. After cleaning five times with PBST buffer, the prepared PVDF membrane was added with 200 μL of ECL ultrasensitive luminescent solution (Solarbio). The automated chemiluminescence image analysis system (Tecan) is used for imaging processing.

### Detection of superoxide anion radical scavenging activity

The analysis of superoxide anion radical (O_2_^−^•) scavenging activity is carried out using a total SOD activity detection kit (Beyotime). Briefly, 20 mL of bacterial cells (OD_600_ = 1.0) was lysed under ultrasound treatment, and 20 μL of bacterial cell lysate containing equal amounts of protein was mixed with 160 μL of WST-8/enzyme working solution. Following 20 μL of reaction starter solution adding and incubating at 37°C for 30 min, the absorbance of the reaction product is measured at 450 nm. O_2_^−^• scavenging activity is calculated according to the instructions.

### ICP-MS assay of intracellular manganese and phosphorus content

The bacterial cells (OD_600_ = 1.0) were washed twice with 50 mM Tris-HCl pH 7.0 buffer containing 10 mM EDTA-Na and washed once with sterile ddH_2_O. The cells were collected, and the cell pellet was freeze-dried using a freeze vacuum dryer. A 0.1 g of freeze-dried bacterial powder was added to 2 mL of nitric acid and dissolved at 160°C for 1 h. After digestion, ultrapure water was added to the solution to make up to 100 mL. The diluent was filtered through a 0.45 µm filter membrane for ICP-MS (iCAP RQ, Thermo Fisher Scientific) measurement.

### Intracellular polyphosphate assay

The intracellular polyphosphate (PolyP) level was detected according to the method as described by Roozbeh et al. (35). Briefly, the fluorescent dye DAPI can bind to PolyP molecules and emit specific yellow-green fluorescence at an excitation wavelength of 415 nm. Fluorescence value at 550 nm can represent the relative content of PolyP inside the bacterial cell. Bacterial cells in Tris-HCl buffer were added with an appropriate amount of DAPI solution (final concentration of 10 μg/mL), incubated at room temperature for 5 min, and fluorescence was detected using a confocal microscope (Leica TCS SP8 STED3X). The confocal microscope images were analyzed using LAS X software (v3.3.0). The relative fluorescence quantification of the PolyP level was measured using SpectraMax M5 Microplate Reader (Molecular Devices).

### Statistical analysis

The experimental data were analyzed using Microsoft Excel (2021) and OriginPro (v10.1.0.178) software. Some results were presented as mean ± standard deviation. Student’s *t*-test was used for significance analysis, where * represents significant differences (*P < 0.05, **P < 0.01, and ***P < 0.001), and ns represents no significant differences.

## Data Availability

All data supporting the findings of this study have been provided in the article and its supplemental material. Correspondence and requests for materials should be addressed to the corresponding author.

## SUPPLEMENTAL MATERIAL

Supplemental material aem.02107-25-s0001.pdf Fig. S1 to S8 and Table S1.

ASM does not own the copyrights to Supplemental Material that may be linked to, or accessed through, an article. The authors have granted ASM a non-exclusive, world-wide license to publish the Supplemental Material files. Please contact the corresponding author directly for reuse.
