# Supplementary material for: DrPitA-mediated enrichment of intracellular manganese and phosphate contributes to oxidative stress resistance of Deinococcus radiodurans
Source: Appl Environ Microbiol. 2026 Apr 21;92(5):e02107-25. doi: 10.1128/aem.02107-25 (PMC13188870; doi:10.1128/aem.02107-25)
Supplement: Supplemental material — Fig. S1 to S8 and Table S1. [file aem.02107-25-s0001.pdf]

Supplemental materials

DrPitA-Mediated Enrichment of Intracellular Manganese and Phosphate Contributes to Oxidative Stress-Resistance of *Deinococcus radiodurans*

Zhenming Xie[a,b], Shang Dai[a,c], Binqiang Wang[a,d,e], Ning Yu[a], Cheng Huang[a], Jie Zhao[a], Chunhui Cai[a], Furong Zhang[a], Zichun Tan[a], Yiting Wang[a], Yuejin Hua[a,f], Bing Tian[a,f*]

a. Institute of Biophysics, College of Life Sciences, Zhejiang University, Hangzhou, China; b. Hangzhou Institute of Medicine (HIM), Chinese Academy of Sciences, Hangzhou, China; c. Department of Microbiology, College of Life Sciences, Nanjing Agricultural University, Nanjing, China; d. State Key Laboratory of Clean Energy Utilization Zhejiang University, Hangzhou, China; e. Zhejiang Baima Lake Laboratory Co., Ltd, Hangzhou, China; f. Cancer Center, Zhejiang University, Hangzhou, China.

* Correspondence: tianbing@zju.edu.cn

866 Yuhangtang Road, Zijingang campus west part, Biophysics Building, Zhejiang University, Hangzhou, 310058, China.

FIG. S1

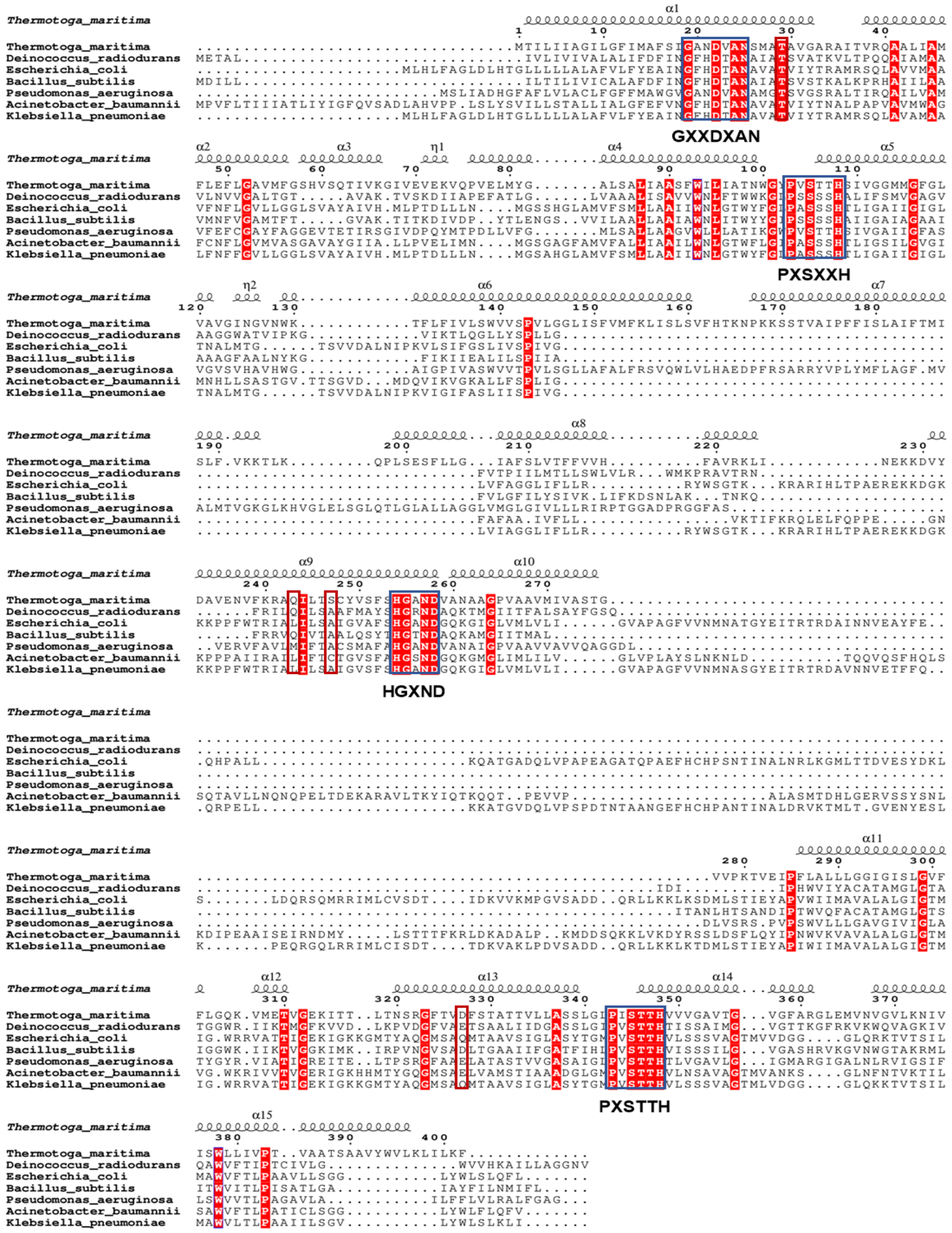

Figure S1. Sequence alignments of PitA homologs from different bacterial species. The sites that may participate in the binding of substrate phosphate and manganese ions are marked with blue and red boxes, respectively.

FIG S2

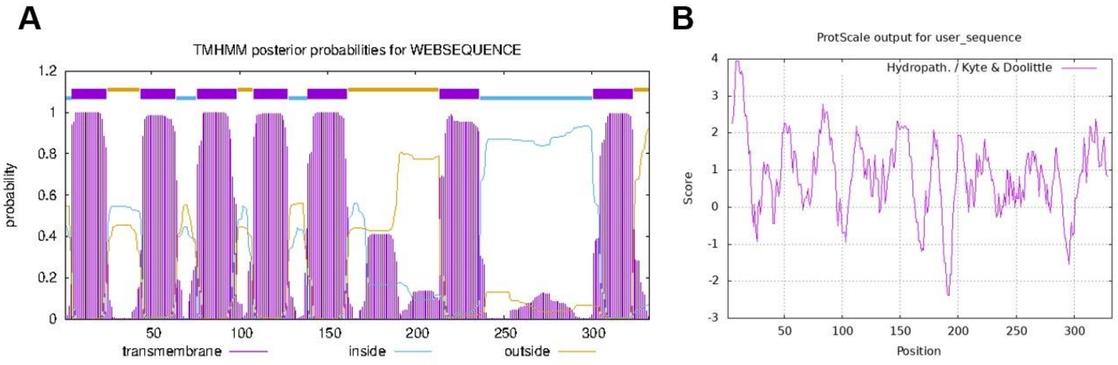

Figure S2. Prediction of transmembrane regions and hydrophobicity analysis of DrPitA protein. (A) DrPitA was predicted containing 10 transmembrane regions. (B) Hydrophobicity analysis indicated that DrPitA has strong hydrophobicity.

FIG S3

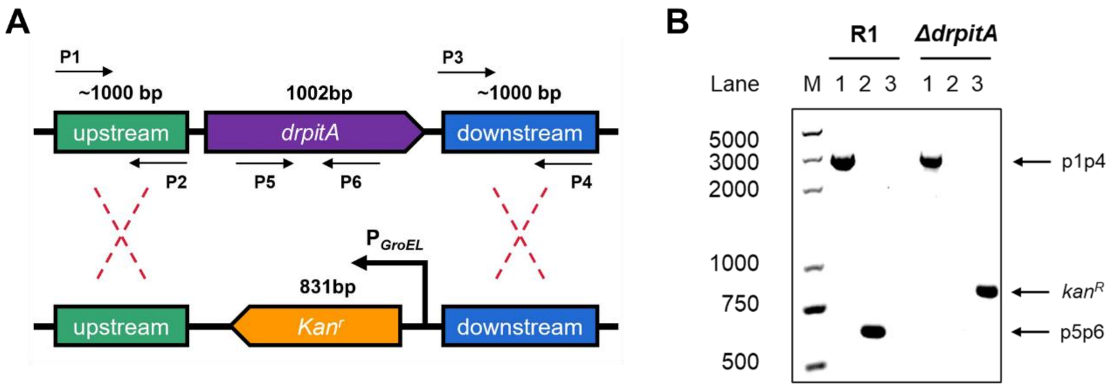

Figure S3. The construction of *drpitA* knockout strain. (A) Schematic diagram of constructing *drpitA* knockout mutant. The target gene was replaced with a kanamycin fragment by homologous recombination; (B) PCR validation of gene mutation using the wild type R1 and knockout strain genomes as PCR templates. PCR amplification was carried out using P1P4 (lane 1), P5P6 (lane 2) and kanamycin resistant fragment (lane 3) primers, respectively, and the obtained PCR products were analyzed by agarose gel electrophoresis.

FIG S4

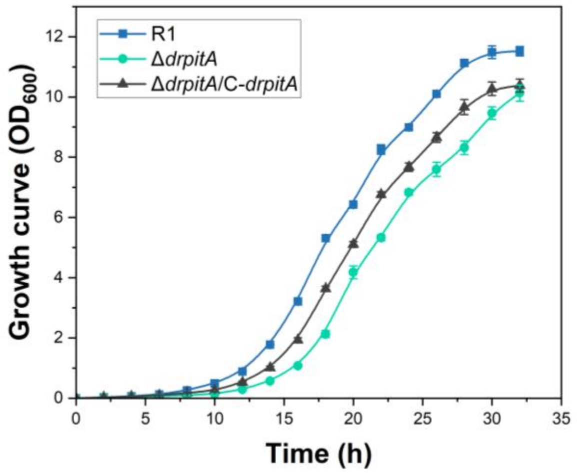

Figure S4 The growth curves of the wild-type, *drpitA* mutant and gene complementation strains in TGY medium.

FIG S5

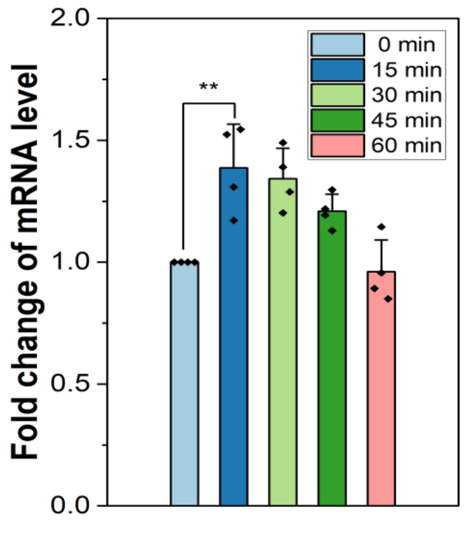

Figure S5. Transcription level analysis of *drpitA* gene under oxidative stress in the wild-type R1.

FIG S6

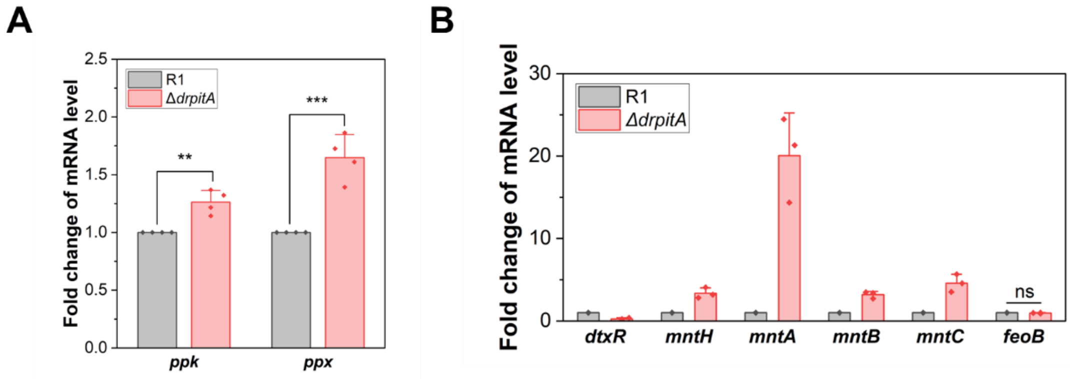

Figure S6. Effects of *drpitA* gene deletion on the transcription levels of polyphosphate metabolism genes (A) and other manganese ion homeostasis related genes (B). The data is presented as mean±standard deviation(SD); ***, *P*<0.001, **, *P*<0.01; ns, not significant.

FIG S7

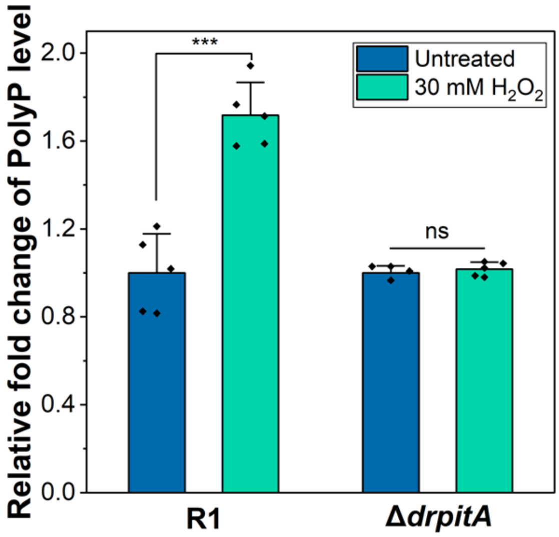

Figure S7. The relative fold change of intracellular PolyP levels measured using fluorescence quantification in the wild-type and mutant strains before and after hydrogen peroxide treatment. ***, *P*<0.001; ns, no significant difference

FIG S8

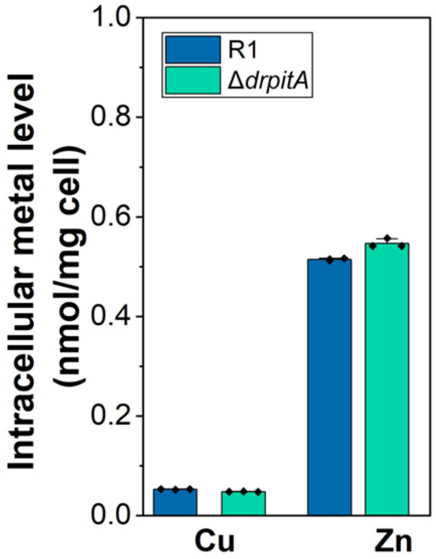

Figure S8. Intracellular copper and zinc ion content in wild-type R1 and Δ*drpitA* mutant grown in TGY medium.

Table S1 Primer sequences used for real-time quantitative PCR (qRT-PCR)

Primer name	Sequence (5`-3`)
dr1343-F	GCGATCAACATCATTCCCACCTC
dr1343-R	CGGCTTCACGGAAGACGTTGTT
dr0925-F	GTGCTGCGCTGGATGAAACCC
dr0925-R	TGTCGATCTGGCTGCCGAAGTAG
dr2539-F	CCGCCGAGGATTACCTCAAGC
dr2539-R	AACAGTTCGAGCAGACGGTGGTG
dr1709-F	CGGCAACTTTGCGACCAACATC
dr1709-R	CACGAGTTCGGCCTGAATCCAGTA
dr2283-F	TGGTGCTGATCGGGCTGGTG
dr2283-R	CGGTGTCGAGGTAATAACTGGCATACA
dr2284-F	TTCCCGTCACGGTCTGGGACA
dr2284-R	GCCAGCATTCGGGCAAGCA
dr2523-F	TCAAGCTGCACGCTGGAGGAGA
dr2523-R	GCGAATCTGGGCCGAGTAAGC
dr1219-F	TGGAAGGTGCTGTGGAAACACG
dr1219-R	GCCAGTCGCCGAAGCCCA
dr1236-F	AAGTTCCTCGCTTACCGCCTGAC
dr1236-R	CGCACTGAGGTATTCGGCCTTG
dr1939-F	TGCTGCACCATCCCTACGACTC
dr1939-R	CGATCATCGCCACGACCTGC
drA0185-F	GGCCGAGCTGTCACGCGA
dr A0185-R	ACGGGCGATCTGGGCAATGA
